# Connections between prolactin and ovarian cancer

**DOI:** 10.1371/journal.pone.0255701

**Published:** 2021-08-06

**Authors:** Amira Alkharusi, Abdullah AlMuslahi, Najwa AlBalushi, Radiya AlAjmi, Sami AlRawahi, Asmaa AlFarqani, Gunnar Norstedt, Fahad Zadjali

**Affiliations:** 1 Department of Physiology, College of Medicine and Health Sciences, Sultan Qaboos University, Muscat, Oman; 2 Department of Pathology, College of Medicine and Health Sciences, Sultan Qaboos University, Muscat, Oman; 3 Department of Women’s and Children’s Health, Karolinska Institutet, Stockholm, Sweden; 4 Department of Biochemistry, College of Medicine and Health Sciences, Sultan Qaboos University, Muscat, Oman; Northwest University, UNITED STATES

## Abstract

Ovarian cancer (OC) is characterized by a high morbidity and mortality, highlighting a great need for a better understanding of biological mechanisms that affect OC progression and improving its early detection methods. This study investigates effects of prolactin (PRL) on ovarian cancer cells, analyzes PRL receptors (PRLR) in tissue micro arrays and relates PRLR expression to survival of ovarian cancer. A database, composed of transcript profiles from OC, was searched for PRLR expression and results were put in relation to survival. Expression of PRLR in OC tissue sections and OC cell lines SKOV3, OV2008 and OVSAHO was assessed using immunohistochemistry, western blots and quantitative real-time PCR. The biological function of PRLR was evaluated by proliferation, colony formation and wound healing assays. Levels of PRLR mRNA are related to survival; in epithelial OC a high PRLR mRNA expression is related to a shorter survival. Analysis of a tissue micro array consisting of 84 OC showed that 72% were positive for PRLR immuno-staining. PRLR staining tended to be higher in OC of high grade tumors compared to lower grades. PRLR mRNA and protein can further be detected in OC cell lines. Moreover, *in vitro* treatment with PRL significantly activated the JAK/STAT pathway. PRLR expression is associated with OC survivals. PRL and its receptor may play an onco-modulatory role and promote tumor aggressiveness in OC. Alternatively, increased PRLR levels may form a base for the development of PRLR antagonist or PRLR antagonist-drug conjugate to increase selective uptake of anti-cancer drugs.

## Introduction

Ovarian cancer (OC) is often diagnosed at an advanced stage and is a major cause of morbidity and mortality in women worldwide with higher mortality compared to other gynecologic malignancies [1, 2]. Its occurrence tends to increase in younger women in recent years [3]. Despite advancements in surgical intervention, chemotherapy and radiotherapy, the survival rate of OC continues to be low and is associated with the tumor stage and the histological tumor type with an overall relative survival of 65%, 44%, and 36% at 2, 5, and 10 years, respectively [4–6]. Serous ovarian carcinoma is the most common subtype of epithelial ovarian carcinoma accounting for 68–71% of all cases followed by endometrioid (9–11%), clear cell (12–13%), mucinous (3%) and transitional (1%) [7]. Most of OC cases are diagnosed in advanced stages due to a high invasive nature, lack of early symptoms, screening strategies and diagnostic markers. Trans-vaginal ultrasound and serum measurement of serum cancer antigen 125 (CA-125) are currently used as a screening test for the high-risk population. However, these screening modalities lacks selectivity toward malignant and benign tumors [8]. The sensitivity of serum CA-125 is less than 60% in early stages and when it is associated with ultrasound imaging, the positive predictive value (PPV) is improved by only 20% [9–11]. Other novel biomarkers for OC have not yet fulfilled criteria of sensitivity and specificity to be approved for clinical application [12].

An emerging role of prolactin (PRL) has been proposed in several different cancers including OC. Elevated serum PRL levels has been observed in all different stages of OC along with CA-125 [13]. Expression of the PRL receptor (PRLR), a member of the cytokine receptor family, is for unknown reasons shown to be high in OC [14]. Beside the well known function of PRL in mammary gland development and lactation, PRL stimulates proliferation of different cell types in the body [15, 16] and has been linked to enhanced tumor cell growth [17]. Increased PRLR levels in tumors may therefore be a sign of increased PRL stimulated cancer cell growth [18, 19]. Recent findings demonstrate that PRL production is not only restricted to the pituitary gland [20], and extra-pituitary PRL production may be of relevance in cancer. In humans and other primates, but not in rodents, research has demonstrated expression of the PRL gene in several extra-pituitary tissues [21, 22]. This is because of the presence of an alternative promoter located 5.8 kb upstream of the pituitary transcription start site which drives extra-pituitary PRL expression [23–26]. Although different promoters control PRL expression in pituitary and extra-pituitary tissues, the human PRL protein sequence is identical regardless of the site of production [27]. Extra-pituitary PRL is similar to the pituitary PRL. The mature form of the 23 kDa PRL protein consists of 199 amino acids in humans, and has four α-helixes arranged in an up-up-down-down style [28]. Other well-characterized 14-, 16-, 22-kDa prolactin protein variants are generated by proteolytic cleavage of the 23kDa protein [28].

Production is well recognized, and although studies indicate a proliferative/anti-apoptotic role for this autocrine/paracrine-produced PRL, the role of extra-pituitary PRL production in humans still requires further investigations [22]. Several publications suggest that activation of the PRL/PRLR signaling pathways is linked to cancer [29] via activation of JAK/STAT [19, 30], PI3K, AKT and MAPK pathways. Despite years of studies, there is still lack of effective diagnostic markers of sufficient sensitivity for clinical applications. There is an emerging need for OC studies to find new diagnostic/prognostic biomarkers and to find new therapeutic targets.

In the present study, we aimed to investigate the role of the PRL/PRLR axis by analyzing effects of PRL on OC cells *in vitro* and by analyzing OC tissue specimens for PRLR expression.

## Materials and methods

### Kaplan Meier plots

Kaplan-Meier survival plots were generated using an online database for ovarian cancers (www.kmplot.com/ovar) [31]. The database contains transcript profiles from epithelial ovarian cancer cases with defined histo-types (serous and endometrioid ovarian cancer). Data base searches were conducted using the search term prolactin receptor (PRLR), then jetset selects the optimal probe set for PRLR gene using a scoring method established to assess each probe set for specificity, coverage and degradation resistance. Based on this we selected the best probes (227629-at) and Kaplan-Meier survival curves, related to PRLR mRNA expression were constructed for epithelial OC for 24 months survival time from 614 high grade- and 381 low grade- epithelial tumors. All cut-off values between upper and lower quartiles were tested and the best threshold was used to split the mRNA expression levels. A more descriptive analysis is described the provide website above.

### Patient specimens and clinical data

Formalin Fixed Paraffin Embedded (FFPE) tissue sections from OC cases were purchased from US Biomax. Tissue Micro Array (TMA) sections with 12 cases of OC and 2 adjacent normal Ovarian (Biomax, USA, cat no. T112b) and TMA section with 72 cases of OC and 2 adjacent normal ovary tissue (Biomax, USA, cat no. BC11012b). Total number of ovarian tissue sections was 84. Breast cancer tissue sections served as a positive control.

### Immunohistochemistry (IHC)

To investigate the expression of PRLR in human OC tissue sections, working conditions were optimized for heat-induced antigen retrieval, after deparaffinization and rehydration of tissue sections as described before [32]. For antigen retrieval, the slides were treated with citrate buffer; pH 6.0, (Nordic Biosite) in pressure cooker for 20 minutes and rinsed in Tris buffered saline containing 0.05% triton (TBST). Endogenous avidin and biotin were neutralized using the Avidin-Biotin Blocking Kit (Dako, USA) and endogenous peroxidase was blocked by 3% H2O2 (Histolab, Stockholm) for 15 minutes at room temperature (RT) in dark. Fc receptor was blocked by treating tissue sections with Fc receptor blocker 30 min, at 20°C (Innovex Biosciences). Then tissue sections were incubated with targeted primary antibody diluted in a common antibody diluting buffer (BioGenix, USA) against PRLR, clone 1A2B1 (Invitrogen, USA) and incubated at 4°C, overnight followed by washing and incubation with biotinylated anti mouse secondary antibody diluted according to the manufacturer’s instructions (BioGenix, USA) for 45 min at room temperature and incubation with streptavidin-biotin-peroxidase complex (BioGenix, USA). Haematoxylin was used for counterstaining. As negative control, the primary antibody was omitted from the staining procedure. Negative controls were performed in parallel to all experiments. IHC stained section analyzed by two independent pathologists at Sultan Qaboos University Hospital and considered the cytoplasmic staining for PRLR positive. The expression of PRLR was scored as described earlier [32]; negative (0% positive cells), mild expression: 1 (<24% positive cells), moderate expression: 2 (>25%-50% positive cells), and high expression: 3 (>50%-75% positive cells).

### Cell lines and cell culture

The ovarian cancer cell lines SKOV3 and OV2008 were obtained from American Tissue Culture Collection (ATCC, USA). OVSAHO cells were established by Prof. Aikou Okamoto, Department of Obstetrics and Gynecology, Jikei University School of Medicine, Tokyo, Japan. SKOV3 cells were cultivated in McCoy′s 5A medium whereas OVSAHO and OV2008 were cultured in RPMI 1640 (Gibco, USA). All cells were cultured with media containing 10% Fetal Bovine Serum (FBS) (Gibco, USA), 100 U/ml penicillin and 100 μg/ml streptomycin (Gibco, USA) in a humidified 5% CO2 incubator at 37°C. Treatment of cells with human recombinant PRL (a generous gift from Novo Nordisk A/S Denmark) was carried out as explained in each experiment. Breast cancer MCF-7 and glioma U251 cells were used as positive controls. All cells were treated with 200 ng/ml PRL. Pilot studies were carried for dose and time dependent experiments to establish the current PRL dose and time.

### RNA extraction and quantitative real-time PCR (qRT-PCR)

RNeasy Mini Kit (Qiagen, USA) was used according to manufacturer´s instructions to extract RNA from lysed cells. Random primers and the High-Capacity cDNA Reverse Transcription Kit (Applied Biosystems, USA) was used to synthesize cDNA from extracted RNA samples. TaqMan Fast Universal PCR Master Mix (Life Technologies, USA) was used to quantify gene expression levels of the samples. The following specific TaqMan probe was used for PRLR (Hs01061477 m1, Thermofisher Scientific). The PCR was performed using a Fast Real-Time PCR system (7900HT, Applied Biosystems, USA). The endogenous control beta-2-microglobulin (B2M) (Life Technologies, USA) was used for normalization and relative expression was determined by the 2-ΔΔCt methods. Three separate experiments were performed. RNA samples from breast cancer (MCF7) and glioblastoma (U251) cells were used as positive control for PRLR expression [19].

### Western blotting (WB)

SKOV3, OVSAHO and OV2008 cells were treated with human recombinant PRL (200ng/mL) for 20 minutes as previously described [19]. Cells were lysed with RIPA buffer (150 mM sodium chloride, 1% NP-40, 0.5% sodium deoxycholate, 0.1% SDS, and 50 mM Tris, pH 8.0). Proteins were separated on a NuPAGE 4–12% Bis Tris gel (Thermofisher Scientific, USA), transferred to a PVDF membrane (Millipore), and blotted with antibodies against PRLR (cat# ab170935, Abcam, UK), STAT3/STAT5 (Cell Signaling, USA) and GAPDH (Sigma Aldrich, Germany) for all cells.

### Alamar Blue assay

Alamar Blue (Invitrogen) was used to test the effect of PRL on proliferation of SKOV3, OVSAHO and OV2008 cells. Standard Alamar Blue protocol was followed. Cells were cultured in 96-well plate (Corning) in serum free media one day before PRL treatment and cells proliferation was assessed after 48 hours exposure to PRL. Thereafter, Alamar Blue was added for three hours and absorbance was read by the Thermo Scientific Multiscan Spectrum at 570/600 nm and compared to the non-treated control cells. The viability of the control cells was set as 100%.

### Colony formation assay

SKOV3 and OV2008 were seeded at a density of 1,000 cells/well and OVSAHO at 5,000 cells/well in a 6-well culture plate (Corning) containing pre-warmed growth media. The plate was gently rotated to uniformly disperse the cells and incubated overnight. Next day, a dose of 200 ng/ml of PRL was added in serum free media to the treated cells. After two days of treatment, media was replaced with growth media and incubation was resumed. Post two weeks, macroscopic clones were carefully washed twice with DPBS (Gibco) and colonies were stained with crystal violet solution for 10–20 min. The staining solution was slowly washed away by immersing the plate in a water-containing pot multiple times. The plate was allowed to air dry and colonies were imaged and counted using Image J (NIH, USA).

### *In vitro* wound healing migration assay

Cells were seeded in 12-well plates (Corning) in triplicates for 24 h and a vertical scratch was introduced by a sterile pipet tip followed by washing to remove un-attached cells. Cells were incubated for 48 hrs with 200ng/ml PRL. The scratch closure was imaged using an inverted light microscope. The analysis of the scratch images was performed by calculating the scratch distance in μm (= open wound distance) for each image. The percentage of healing distance was plotted over time for each cell type before and after treatment. Data are presented as mean ± SE.

### Statistical analysis

Cell culture experiments were performed in triplicates in at least three independent experiments. Statistical significance of the differences was evaluated using unpaired, 2-tailed Student’s *t*-test, ANOVA test with post-hoc analysis, and was considered significant when the significance level of the test was p < 0.05.

## Results

### Increased PRLR expression associated with short survivals in OC patients

**Fig 1:**
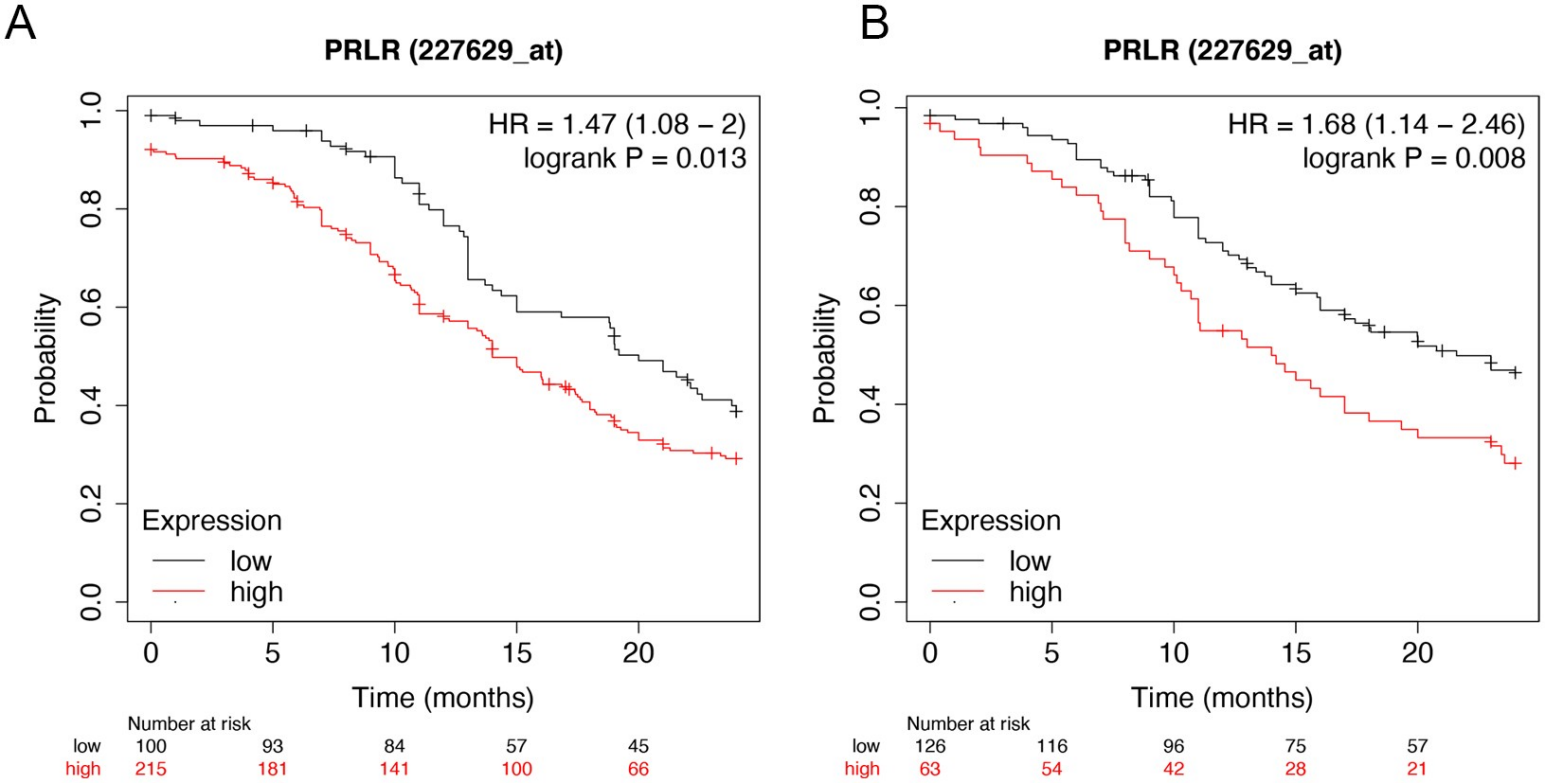
Kaplan Maier survival curve based on PRLR mRNA expression.

Different PRLR markers of OC prognosis have been identified using literature search. Best jet set probes for gene expression analysis of PRLR was evaluated using a website (www.kmplot.com/ovar). A scoring method, established by this website, was used to assess each probe set for specificity, coverage and degradation resistance. Based on this we selected (227629-at) as the best probe. This probe (227629-at) was used to relate PRLR expression to survival using Kaplan-Meier curves. The data consisted of epithelial OC with up to 24 months survival time from 614 high grade- and 381 low-grade epithelial tumors (Fig 1). The survival plots depict lower progression-free survival with higher expression of PRLR in high grade tumor (Hazard ratio = 1.47, P-value <0.05) and low grade tumor (Hazard ratio = 1.68, P-value <0.05) as illustrated in (Fig 1A and 1B).

A Kaplan-Meier plots were generated using an online database for ovarian cancers (www.kmplot.com/ovar). Transcript profiles from 614 high grade- and 381 low grade- epithelial ovarian cancers were examined. The expression of PRLR mRNA related to survival is shown in the figure in the form of Kaplan-Meier survival curves. Fig 1A and 1B shows the survival plots depict lower progression-free survival with higher expression of PRLR in high grade tumor (Hazard ratio = 1.47, P-value <0.05) and low grade tumor (Hazard ratio = 1.68, P-value <0.05). Obviously patient’s survival decrease with increased PRLR expression.

### PRLR expression in ovarian cancer cells

**Fig 2:**
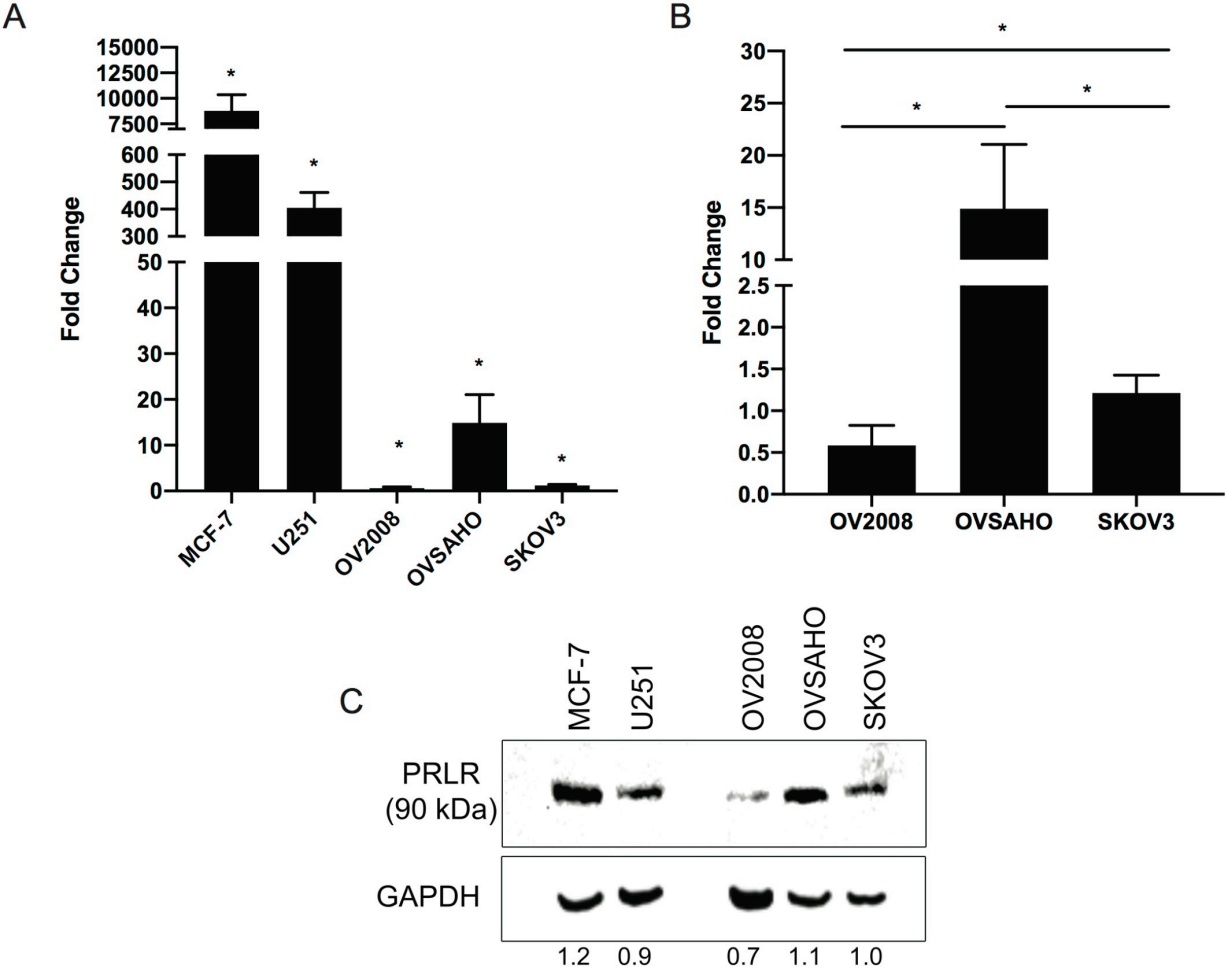
Increased PRLR transcript and protein expressions in ovarian cancer (OC) cells. **A)** *PRLR* mRNA expression levels in 3 ovarian cancer cell lines SKOV3, OVSAHO and OV2008 and two positive control cells; breast cancer cells (MCF7) and brain tumours cells (U251). **B)** Subsection of Fig 2A, to show relative expression between the three OC cell lines. Gene expression levels are reported as fold change with reference to the expression in SKOV3 cells. Results are representative of three separate experiments. Data is presented as mean ± SD (n = 3). Statistical significance is indicated as *P < 0.05. **C)** PRLR protein expression in OC cell lines and the positive controls. The long isoform of PRLR was expressed at 90kDa with highest protein expression in the OVSAHO cells.

We examined the PRLR transcript levels in three different OC cell lines SKOV3, OVSAHO and OV2008 cells. MCF7 and U251 cells were used as positive controls [19]. PRLR mRNA was 500–8000 fold higher in MCF-7 and U251 cells, p-value < 0.05, compared to OCs. In OC, PRLR mRNA expression was relatively higher in OVSAHO compared to the other OC cell lines, p-value < 0.05 (Fig 2A and 2B). PRLR protein was detected in all OC cell lines (Fig 2C). PRLR protein findings are in line with the mRNA expression levels.

### PRLR expression in human OC tissue sections

**Fig 3:**
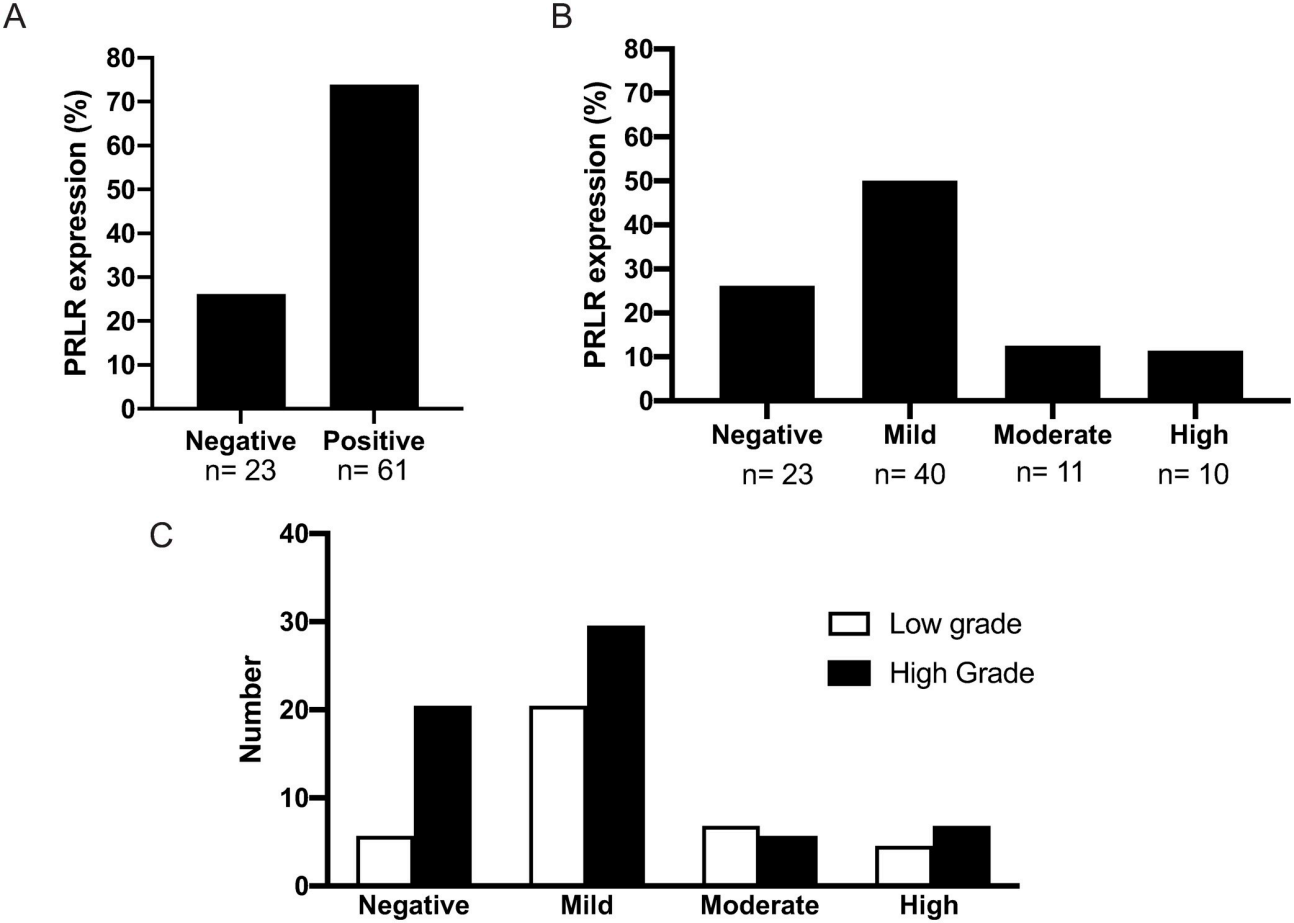
Prolactin receptor immuno-reactivity in ovarian cancer tissues. Histological analyses of a tissue microarray (TMA) with 84 ovarian cancer (OC) and normal ovarian tissues as a control. **A)** PRLR immuno reactivity was detected in 61 out of 84 OC patients (72%) while 23 patients were negative (27%). **B)** PRLR staining in OC tissues; a mild level of PRLR staining (<25% of cells) was detected in 44 out of 84 patients (50%), a moderate level (25–50% of cells) was found in 11 patients (12%) and a high level (>50% of cells) was fond in 10 patients (11%). **C)** Percentages of PRLR positive cells were also shown in OC patients with moderate and high-grade tumour. Mild PRLR staining was detected in 30% of high-grade tumors and 20% of low-grade tumors, moderate level in 6% of high-grade tumors and 7% of low-grade tumors and high-level staining level was found in 7% of high-grade tumors and 5% of low-grade tumors. All percentages were obtained by two independent pathologist and presented with 95% confidence intervals.

**Fig 4:**
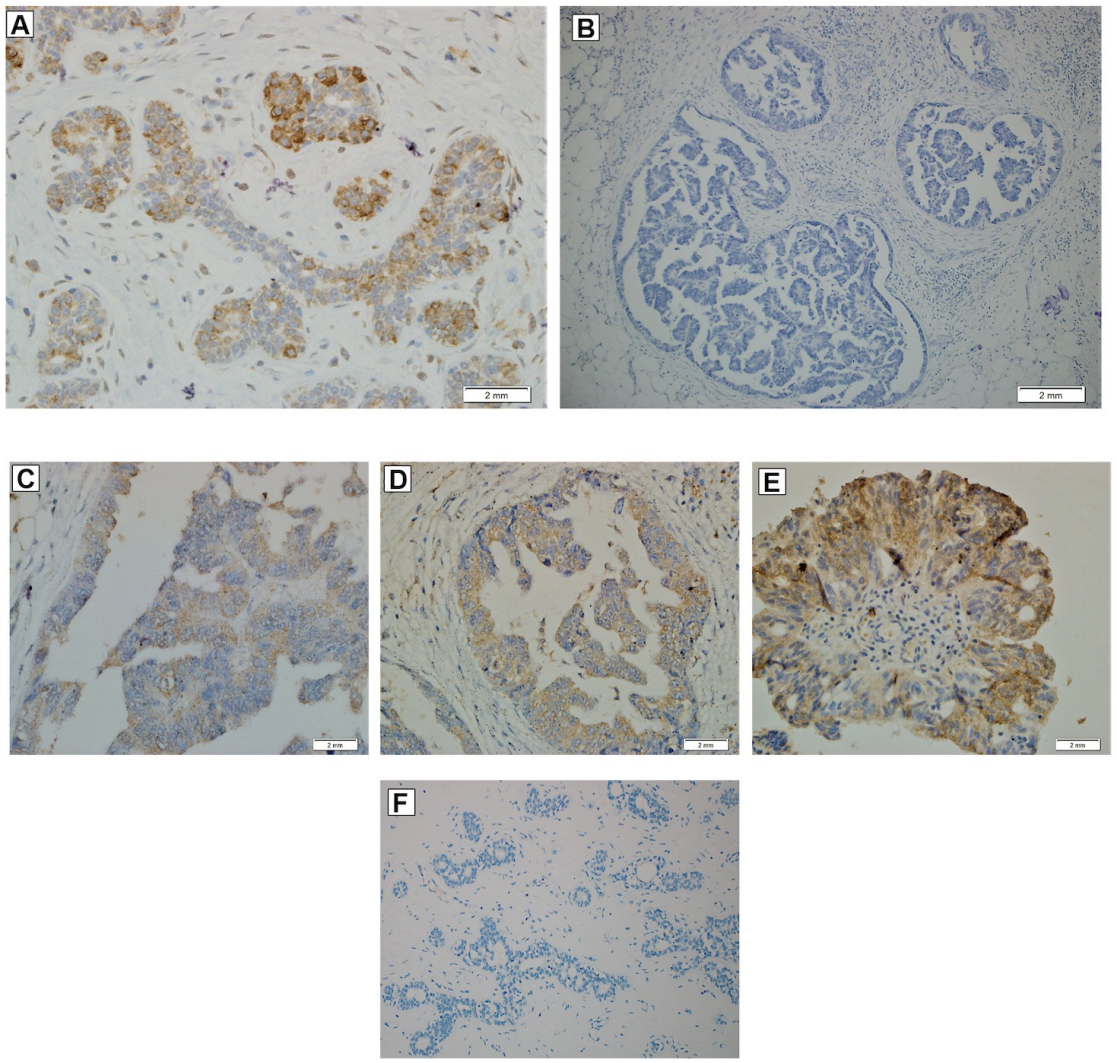
Immuno-histochemical staining using the PRLR antibody in ovarian cancer tissue. **(A)** Breast tissue section shows moderate intensity of cytoplasmic staining with PRLR antibodies and was used as positive control. **(B)** OC tissue section with negative staining for PRLR expression. Grade of staining in OC tissue sections according to staining percentage, **(C)** Mild (<25% of the section area) level **(D)** Moderate (25–50% of the section area) level **(E)** High-grade (>50% of the section area) level. **(F)** Breast tissue section with negative staining for PRLR expression.

Tissue microarrays (TMAs) consisting of 84 tissue samples from OC patients were analyzed by immuno-histochemistry to detect expression of PRLR (Fig 3). Two independent pathologists analyzed the tissue sections for detection of expression and also categorized expression intensity as mild, moderate or high. Out of 84 samples on each tissue sections, 23 samples were negative (27%) and 61 were positive (72%) for PRLR (Fig 3A). Forty samples have mild (<25% of total area) expression of PRL (50%), 11 had moderate (25–50% of the section area) expression (12.5%) and 10 had high (>50% of area) expression (11.4%), (Fig 3B). Expression of PRLR was also detected in all examined adjacent normal ovarian tissue sections at a low level (<25%). We then assessed PRLR staining pattern in low- and high-grade OC tumors (Fig 3C). Mild staining was detected in 29.5% of high-grade tumors and 20.5% of low-grade tumors. Moderate PRLR staining was detected in 5.6% and 6.8% in high- and low-grade tumors, respectively. In high-grade tumors, 6.8% had a high PRLR staining and 4.5% low staining. Examples of tissues with different grades of PRLR staining are shown in (Fig 4).

### PRL induces STAT3/STAT5 phosphorylation in ovarian cancer cells

**Fig 5:**
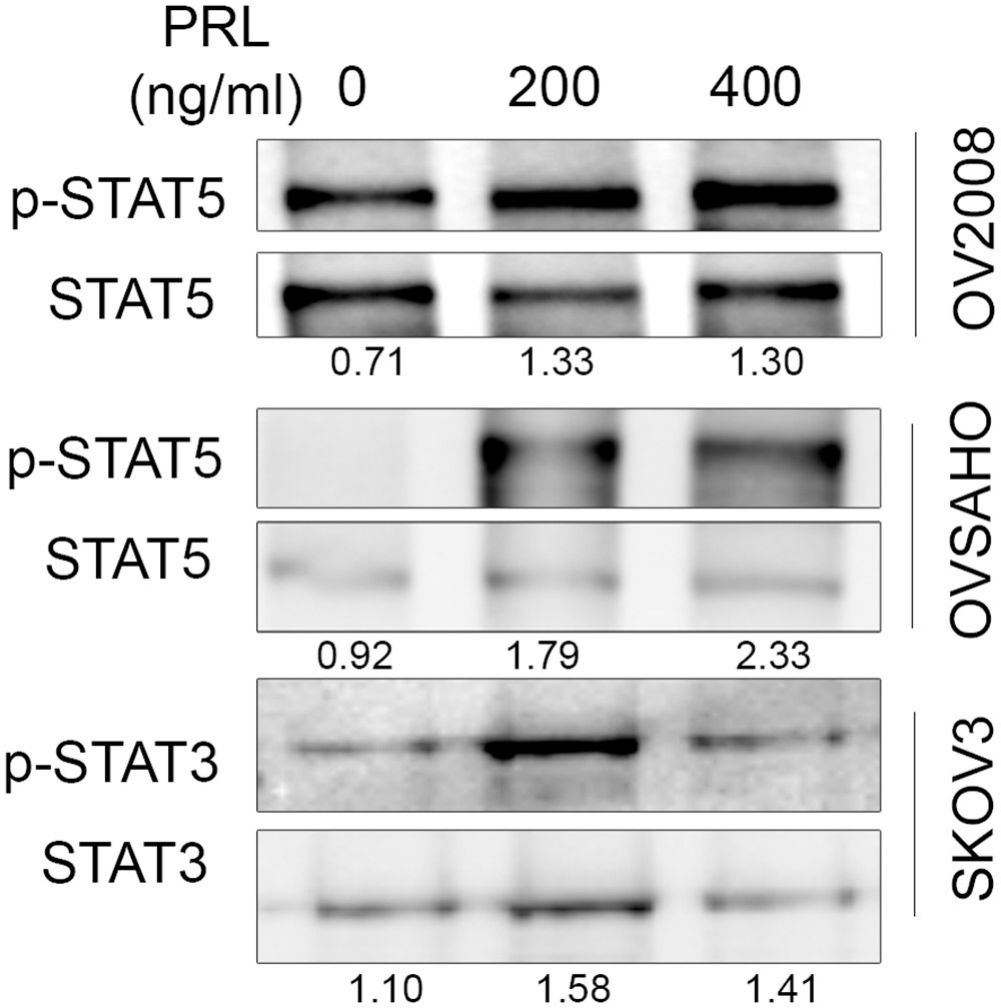
Prolactin signaling in ovarian cancer cells. All cells were kept under serum free conditions overnight and later exposed to 200 and 400 ng/ml human PRL or to control PBS for 20 mints. Following protein extraction and gel electrophoresis phospho- and total STAT5, phospho- and total STAT3 were analyzed using Western blotting. A representative image selected from three independent experiments is shown.

The short-term effect of PRL on intra cellular protein phosphorylation was tested in OC cells (Fig 5). OC cells were treated with two concentrations of PRL (200 and 400 ng/ml) for 20 minutes. OV2008 cells showed constitutively active STAT5 phosphorylation without PRL and this activity increased with PRL treatment. PRL clearly increased STAT5 phosphorylation in OVSAHO cells (Fig 5). STAT5 levels were low in SKOV3 cells and we could not demonstrate any PRL effect on STAT5 phosphorylation. However, when STAT3 activity was measured in SKOV3 cells we could demonstrate increased STAT3 phosphorylation using 200 ng/ml of PRL (Fig 5) while no STAT3 phosphorylation was detected in OV2008 and OVSAHO cells.

### Prolactin promotes migration and colony formation but not proliferation of OC cells

**Fig 6:**
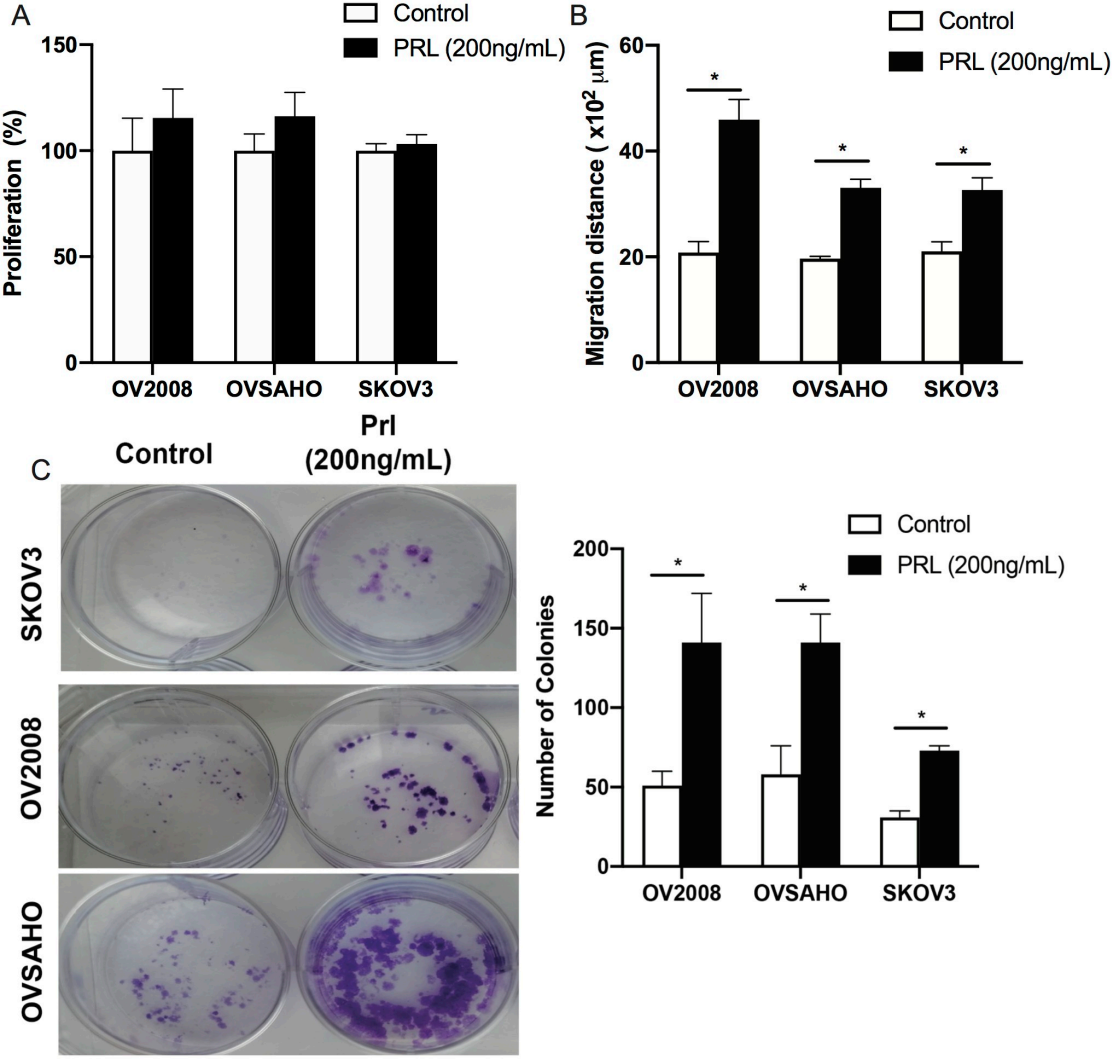
Effect of PRL on biological functions of ovarian cancer cells **A)** Effect of PRL on OV2008, OVSAHO, and SKOV3 cell proliferation. Proliferation was assessed by Alamar Blue staining after 48 hours treatment with 200 ng/ml human PRL. **B)** Effect of PRL on migration of ovarian cells using so called scratch assay or wound healing assay. OV2008, OVSAHO, and SKOV3 cells were cultured with 200 ng/ml PRL under serum free conditions in 12 well plates for 48 h. The migratory capacity of all cells was measured by measuring the healing distance in μm. **C)** Colony forming ability of OC cell lines using colony formation assay. Results represent the means ± SE from three independent experiments. A representative photo image is shown for colonies. All data were analysed by one-way ANOVA followed by Tukey post-hoc test. * = P-value <0.05.

To further investigate the functional effect of PRL on the biological behavior of OC cells, proliferation, migration and colony formation assays were conducted. PRL treatment for 48 hours did not induce proliferation of OC cells under the test conditions used. However, there was a significant increase in the migration of the three cell types after 48 hrs of PRL treatment (p-value<0.05), (Fig 6A and 6B). The colony formation rate was assessed after PRL treatment in all cell lines (Fig 6C). PRL significantly increased the colony formation rate in all cell types. These findings indicated that PRL affects the biological behavior of OC cells.

## Discussion

In this study we extracted data on epithelial OC cases and calculated the PRLR expression in relation to survival using Kaplan-Meier survival curves. We found that high PRLR expression in OC is associated with shorter survivals. By using immuno-histochemistry we further demonstrated PRLR expression in OC tissue sections. PRLR expression in cultured OC cells is detectable both at mRNA and protein levels and PRL treatment of cultured OC cells increased cell migration and colony formation rates.

Two different pathological approaches were used for immuno-histochemical analysis of tissue sections, namely staining intensity and percentage of stained malignant cells. Both approaches revealed heterogenous expression of PRLR in OC sections. More than 50% of OC tissue sections expressed PRLR at a low level using either of the pathological approaches. The remaining tissue sections varied from moderate to high expression. A marked and heterogenous elevation in PRLR was previously reported in other types of tumors including breast cancer [33], prostate cancer [34], hepatocellular carcinoma [35] and glioblastoma multiforme [19].

Analysis of OC sections with high grade or low grade tumors, showed diverse expression in both grades. Negative PRLR expression was seen mostly in high-grade OC tumors but the tissue array had fewer (33 cases out of 84) of low-grade samples. No significant association was detected between PRLR expression levels and OC tumor grade (P-value >0.05). Such a non-significant association has been reported in other cancers such as hepatocellular carcinoma [27] and breast cancer [36]. When a database consisting of OC transcript profiles were used to relate PRLR expression to survival it was evident that PRLR expression is associated to survival rate. It is here relevant to point out that OC exist in different forms and further analysis could better related PRLR expression to different forms of OC. Analysis of OC sections against STAT3/STAT5 were not done in this study but previous supported showed increased STATs expressions in OC tissues. In ascites derived OC cells, increased p-STAT3 levels were detected in 90–95% of the patients [37]. Also, expression of p-STAT3 and p-STAT5 but not p-STAT1 and p-STAT6, were significantly higher in ovarian cancer cells compared to benign and normal tissues [38]. Similar findings were also observed in p-STAT3 and total STAT3 in ovarian cancer tissues compared to benign and normal tissues [39].

In addition to the above findings, we found detectable levels of PRLR, mRNA and protein levels in three different OC cell lines and at a concentration of 200 ng/ml, PRL was shown to promote JAK/STAT activation in all OC cells. The STAT3/5 regulation by PRL observed in this study supports previous studies showing activation of these transcription factors in different types of cancer cells and the effect is cell-dependent [19, 29, 32]. It had been also shown that PRL induces production of different cytokines in murine peritoneal macrophages, including interleukin-1β (IL-1β), interleukin-12β (IL-12β), interferon-γ (IFN-γ), and TNF [40]. An autocrine loop of PRL signaling may enhance the inflammatory response of activated monocytes [41]. According to previous studies, the concept of locally produced PRL, which can activate its receptor on target cells, has emerged as a new mechanism in chronic inflammatory diseases and several hormone sensitive cancer models [42–44].

STAT5 is a transcription factor that is activated by PRL and phosphorylation of this protein has an anti-apoptotic role in cells. Previous studies have shown that phosphorylated STAT5 mediate oncogenic effects of EGFR in different tumor tissues [45–47]. In this study, OV2008 cell line showed constitutive STAT5 activation and this is because OV2008 cells are an ovarian cancer cells shown to have constitutive active pathways like PTEN, mTOR and Akt [48, 49]. This could be explained the p.E545K mutation in PIK3CA gene; this variant lies within the helical domain of the p110α protein that is linked a constitutive active signaling pathways [50, 51]. Collectively, all these findings support a concept that PRL and its receptor can have a significant role in OC pathogenesis.

Furthermore we showed increased migration and colony formation properties of OC cells with PRL treatment. However, in our hands PRL is not a strong mitogenic factor by itself and this seems to be the case also in our previous studies concerning glioblastoma [19] and leiomyoma cells [52]. It is fair statement that the precise role of PRL and its receptor in tumor biology is not yet clear but the present results taken together with previous investigations support a role for PRL/PRLR as being an onco-modulatory factor. Findings of increased PRLR levels in tumors combined with a frequently observed elevation of serum PRL in cancer patients formed the basis for a clinical trial to suppress serum PRL with dopamine receptor agonists. However, such treatment failed to demonstrate significant effects on tumor growth [53]. Moreover, due to the more recent demonstration of PRL production outside the pituitary gland, attempts have been made to block PRL by using PRLR antagonists (PRLRA) [54]. It has been shown that PRL variants acting as competitive PRLR antagonists can efficiently down regulate PRLR signaling, cell survival, and/or proliferation in various breast or prostate cancer preclinical assays [43, 44]. Moreover, PRL mRNA expression in the synovial tissue of rheumatoid arthritis patients positively correlates with several clinical disease parameters, which confirm the local PRL effect on disease pathogenesis.

A PRLR blocking monoclonal antibody showed significant antitumor activity against MCF7 breast cancer xenografts [55]. Our previous study in glioblastoma demonstrated increased PRLR expression levels and indicated some efficacy to reduce tumor cell growth by using PRLRA in experimental systems. Although a phase I clinical trial with PRLR neutralizing antibodies (LFA102) in breast or prostate cancer patients showed no antitumor activity when used as monotherapy [56], a lot of studies supported the rationale of using this approach in chronic inflammatory diseases like rheumatoid arthritis [57].

Future studies are needed to better clarify roles of PRL in different types of tumors and to explore the potential use of PRLRA as a novel therapeutic approach in different disease models. In conclusion, the current study showed that PRL and its receptor exert an onco-modulatory role in OC and blocking this receptor suggests a new line of anticancer treatments. Alternatively, increased PRLR levels may form the bases for the creation of drugs conjugated to PRLR antagonists to increase selective uptake of anti-cancer drugs.

## Supporting information

S1 Fig (PDF)
