# Supplementary figures and images for: Connections between prolactin and ovarian cancer
Source: PLoS One. 2021 Aug 6;16(8):e0255701. doi: 10.1371/journal.pone.0255701 (PMC8345882; doi:10.1371/journal.pone.0255701)

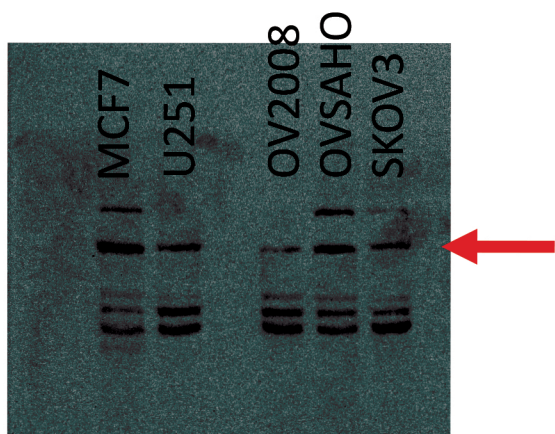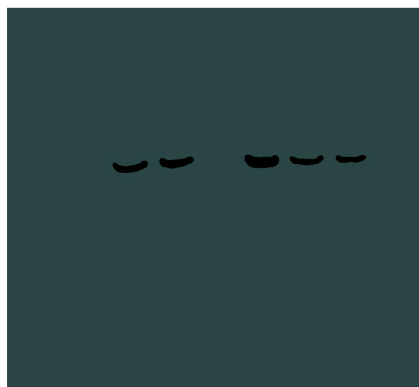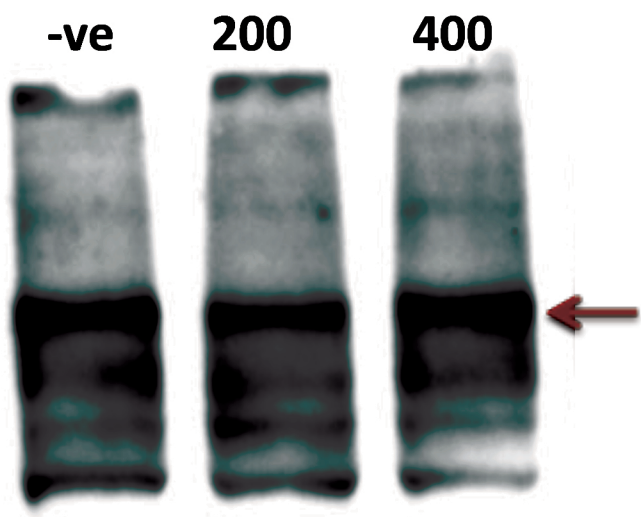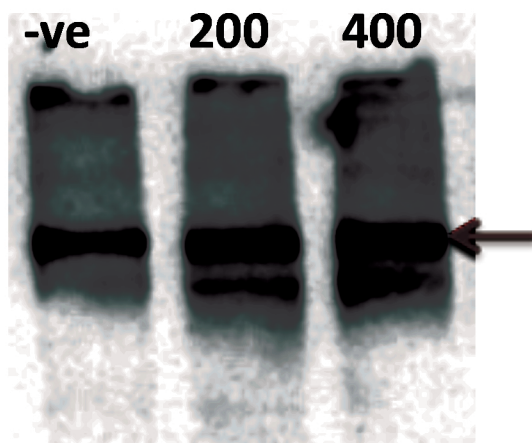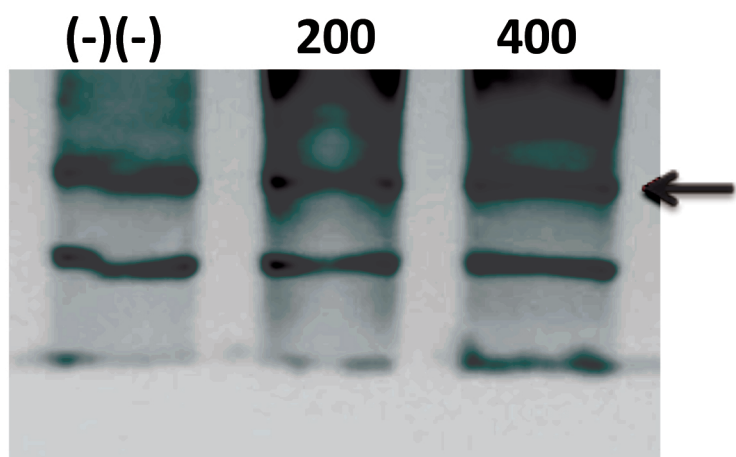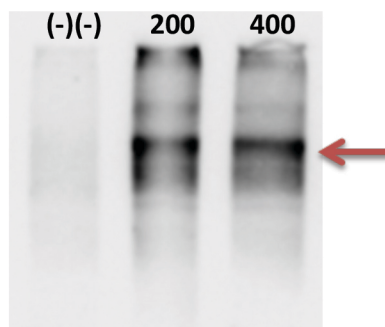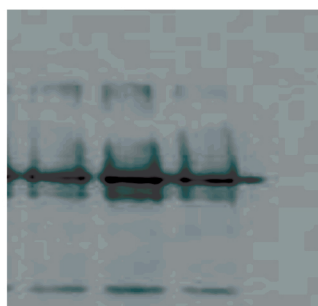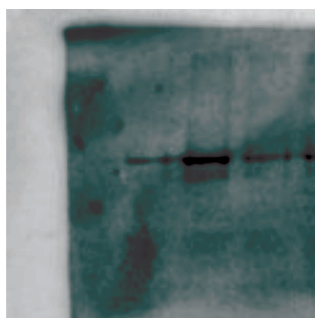

Supplement: S1 Fig — (PDF) [file pone.0255701.s001.pdf]
